# Fracture and Bone Mineral Density Response by Baseline Risk in Patients Treated With Abaloparatide Followed by Alendronate: Results From the Phase 3 ACTIVExtend Trial

**DOI:** 10.1002/jbmr.3848

**Published:** 2019-09-11

**Authors:** Benjamin Z Leder, Carol Zapalowski, Ming‐Yi Hu, Gary Hattersley, Nancy E Lane, Andrea J Singer, Robin K Dore

**Affiliations:** ^1^ Harvard Medical School Boston MA USA; ^2^ Radius Health, Inc. Waltham MA USA; ^3^ School of Medicine University of California at Davis Sacramento CA USA; ^4^ Medstar Georgetown University Hospital Washington DC USA; ^5^ David Geffen School of Medicine University of California at Los Angeles Los Angeles CA USA

**Keywords:** ABALOPARATIDE, BASELINE RISK SUBGROUPS, BONE MINERAL DENSITY, FRACTURE PREVENTION, POSTMENOPAUSAL OSTEOPOROSIS

## Abstract

In the randomized, placebo‐controlled, double‐blind phase 3 ACTIVE study (NCT01343004), 18 months of abaloparatide 80 μg daily (subcutaneous injection) in postmenopausal women at risk of osteoporotic fracture significantly reduced the risk of vertebral, nonvertebral, clinical, and major osteoporotic fractures and significantly increased bone mineral density (BMD) versus placebo regardless of baseline risk factors. Women from the abaloparatide and placebo groups who completed ACTIVE were eligible for ACTIVExtend (NCT01657162), in which all enrollees received sequential, open‐label monotherapy with alendronate 70 mg once weekly for up to 24 months. This prespecified analysis evaluated whether fracture risk reductions and bone mineral density (BMD) gains associated with abaloparatide during ACTIVE persisted through the full 43‐month ACTIVE–ACTIVExtend study period in nine prespecified baseline risk subgroups. Baseline risk subgroups included BMD *T*‐score at the lumbar spine, total hip, and femoral neck (≤ − 2.5 versus > − 2.5 and ≤ −3.0 versus > − 3.0), history of nonvertebral fracture (yes/no), prevalent vertebral fracture (yes/no), and age (<65 versus 65 to <75 versus ≥75 years). Forest plots display treatment effect. Treatment‐by‐subgroup interactions were tested using the Breslow‐Day test, Cox proportional hazards model, and ANCOVA model. After the combined ACTIVE–ACTIVExtend study period, reductions in relative risk for new vertebral, nonvertebral, clinical, and major osteoporotic fractures were greater among patients in the abaloparatide/alendronate group than among those in the placebo/alendronate group across all nine baseline risk subgroups. BMD gains at the lumbar spine, total hip, and femoral neck were greater in the abaloparatide/alendronate group versus the placebo/alendronate group. No clinically meaningful interaction between treatment assignment and any baseline risk variable was observed. The sequence of abaloparatide for 18 months followed by alendronate for up to 24 months appears to be an effective treatment option for a wide range of postmenopausal women at risk for osteoporotic fractures. © 2019 The Authors. *Journal of Bone and Mineral Research* published by Wiley Periodicals, Inc.

## Introduction

Abaloparatide for subcutaneous injection is a human parathyroid hormone–related peptide [PTHrP(1‐34)] analog available for the treatment of postmenopausal women with osteoporosis who are at high risk for fracture.^1^ The efficacy and safety of abaloparatide were evaluated in a phase 3, multicenter, double‐blind, randomized, placebo‐ and active‐controlled trial (the Abaloparatide Comparator Trial in Vertebral Endpoints: ACTIVE, NCT01343004) that enrolled 2463 postmenopausal women at high risk for osteoporotic fracture.^2^ Treatment with abaloparatide 80 μg daily for 18 months was associated with significantly greater reductions in the incidences of new vertebral, nonvertebral, clinical, and major osteoporotic fractures versus placebo (all *p* < 0.05). In addition, bone mineral density (BMD) gains were significantly greater with abaloparatide versus placebo at total hip, femoral neck, and lumbar spine at all time points evaluated (6, 12, and 18 months; all *p* < 0.001).

Analysis of prespecified subgroups from ACTIVE demonstrated that fracture risk reductions and increases in BMD were consistent across a wide variety of baseline risk factors, including BMD *T*‐scores at lumbar spine, total hip, and femoral neck, age, prevalence of vertebral fracture, history of nonvertebral fracture, and baseline fracture risk as assessed by FRAX.^3^, ^4^

At the end of ACTIVE, patients from the abaloparatide and placebo groups were given the opportunity to enroll in an extension study, ACTIVExtend (NCT01657162), to receive 24 months of sequential antiresorptive treatment with alendronate. Results from the full 43 months of the integrated ACTIVE‐ACTIVExtend (18 months of abaloparatide or placebo in ACTIVE, with about 1 month off treatment for re‐consent, followed by 24 months of alendronate treatment) have been published.^5^ In summary, benefits associated with abaloparatide treatment in ACTIVE were maintained through ACTIVExtend with subsequent alendronate treatment. The incidence of new vertebral fractures was 5.6% in the placebo followed by alendronate (placebo/alendronate) group compared with 0.9% in the abaloparatide followed by alendronate (abaloparatide/alendronate) group, a relative risk reduction of 84% (*p* < 0.001). There were also risk reductions of 39%, 34%, and 50% in nonvertebral fractures, clinical fractures, and major osteoporotic fractures, respectively, in the abaloparatide/alendronate group versus the placebo/alendronate group (all *p* < 0.05). Additionally, BMD gains achieved during abaloparatide treatment in ACTIVE were maintained in ACTIVExtend.

The primary objective of this analysis was to evaluate whether the fracture risk reductions and the BMD gains observed with abaloparatide/alendronate in ACTIVExtend were consistent across prespecified subgroups categorized by baseline risk.

## Materials and Methods

In ACTIVE, patients were randomized 1:1:1 to placebo or abaloparatide 80 μg daily in a blinded fashion or to open‐label teriparatide 20 μg daily for 18 months.^2^ The design and methodology of ACTIVExtend (NCT01657162) have been described in detail.^5^ ACTIVExtend was conducted in accordance with the Declaration of Helsinki and in compliance with Good Clinical Practice guidelines and all other applicable local regulatory and ethical requirements.

A total of 581 and 558 eligible patients who completed ACTIVE entered ACTIVExtend from the placebo and abaloparatide groups, respectively, and comprised the intent‐to‐treat (ITT) population. All patients in ACTIVExtend received oral alendronate 70 mg once weekly for up to 24 months. Patients with evaluable spinal radiographs at ACTIVE baseline and at the end of ACTIVExtend (month 43) comprised the modified ITT population (mITT: *n* = 568 and *n* = 544 in the placebo/alendronate and abaloparatide/alendronate groups, respectively) for evaluation of new vertebral fracture. New vertebral, nonvertebral, clinical, and major osteoporotic fracture incidence and BMD changes at the lumbar spine, total hip, and femoral neck over the entire 43‐month period from ACTIVE baseline to the end of ACTIVExtend were evaluated.

Prespecified subgroup analyses included evaluation of the following nine ACTIVE baseline risk subgroups:

- Lumbar spine BMD *T*‐score: ≤ − 2.5 versus > − 2.5 and ≤ −3.0 versus > − 3.0
- Total hip BMD *T*‐score: ≤ − 2.5 versus > − 2.5 and ≤ −3.0 versus > − 3.0
- Femoral neck BMD *T*‐score: ≤ − 2.5 versus > − 2.5 and ≤ −3.0 versus > − 3.0
- Nonvertebral fracture history: yes or no
- Prevalent vertebral fracture: yes or no
- Age: <65 versus 65 to <75 versus ≥75 years

### Statistical analysis

Subgroup analyses were performed for efficacy endpoints through 43 months for each prespecified subgroup. Relative risk ratio and 95% confidence interval (CI) for the treatment difference in the new vertebral fracture endpoint were determined for each subgroup using the mITT population; treatment‐subgroup interactions were tested with the Breslow‐Day test. Hazard ratios and 95% CI were determined for nonvertebral, clinical, and major osteoporotic fractures using the ITT population; treatment‐subgroup interactions were assessed using Cox proportional hazards models. Lumbar spine, total hip, and femoral neck BMD percent changes were calculated as least squares mean differences; treatment‐subgroup interactions were assessed by an analysis of covariance model with last observation carried forward (LOCF). No *p* value adjustments were made for multiple comparisons.

## Results

Patient characteristics in ACTIVExtend at baseline were representative of the overall population of ACTIVE. Abaloparatide/alendronate and placebo/alendronate subgroups were well matched for baseline characteristics, such as age, lumbar spine, total hip, and femoral neck BMD *T*‐scores, and prior vertebral and nonvertebral fracture (Table 1).

**Table 1:** Predefined Baseline Risk Subgroups in ACTIVExtend: ITT Population

	Placebo/alendronate	Abaloparatide/alendronate
All participants, *n* (%)	581 (100.0)	558 (100.0)
*T*‐score categories^a^		
Lumbar spine, *n* (%)		
≤ − 2.5	442 (76.1)	410 (73.5)
> − 2.5	139 (23.9)	148 (26.5)
≤ − 3.0	305 (52.5)	293 (52.5)
> − 3.0	276 (47.5)	265 (47.5)
Total hip, *n* (%)		
≤ − 2.5	139 (23.9)	121 (21.7)
> − 2.5	442 (76.1)	437 (78.3)
≤ − 3.0	41 (7.1)	37 (6.6)
> − 3.0	540 (92.9)	521 (93.4)
Femoral neck, *n* (%)		
≤ − 2.5	169 (29.1)	158 (28.3)
> − 2.5	412 (70.9)	400 (71.7)
≤ − 3.0	56 (9.6)	45 (8.1)
> − 3.0	525 (90.4)	513 (91.9)
Fracture status^a^		
Prevalent vertebral fracture at baseline^a^^,^ b		
Yes	132 (22.8)^c^	123 (22.0)
No	448 (77.2)^c^	435 (78.0)
At least 1 prior nonvertebral fracture^d^^,^ e		
Yes	282 (48.5)	272 (48.7)
No	299 (51.5)	286 (51.3)
Age categories		
<65 years	114 (19.6)	106 (19.0)
65 to <75 years	370 (63.7)	351 (62.9)
≥75 years	97 (16.7)	101 (18.1)

ITT = intent to treat.

a Based on ACTIVE study baseline.

b Evaluated by BioClinica‐Synarc.

c Percentages are based on *n* = 580.

d Based on fractures that occurred before visit 3 in ACTIVE recorded on the “Clinical Fractures” case report form (CRF) page.

e Excluded those of spine, breastbone, kneecap, toes, fingers, or skull and facial bones.

**Figure 1:**
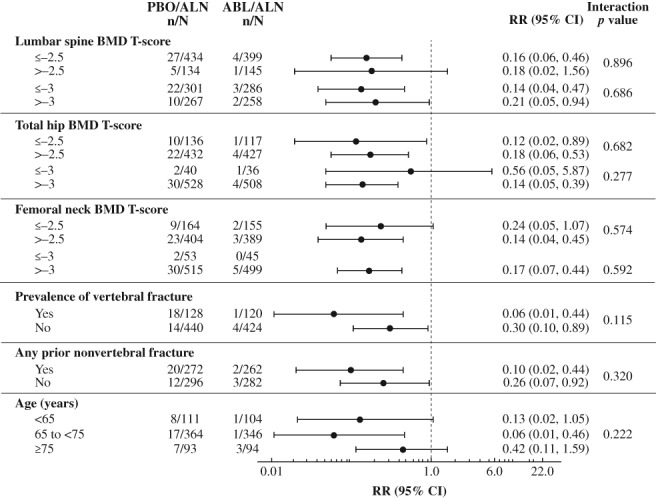
New vertebral fracture risk reduction for abaloparatide/alendronate versus placebo/alendronate by prespecified subgroup. ABL = abaloparatide; ALN = alendronate; BMD = bone mineral density; CI = confidence interval; mITT = modified intent‐to‐treat; PBO = placebo; RR = risk ratio.

**Figure 2:**
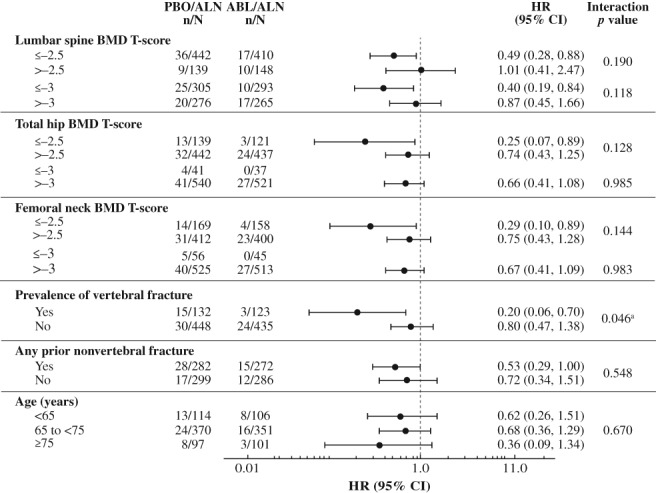
Nonvertebral fracture risk reduction for abaloparatide/alendronate versus placebo/alendronate by prespecified subgroup. ABL = abaloparatide; ALN = alendronate; BMD = bone mineral density; CI = confidence interval; HR = hazard ratio; PBO = placebo. ^a^No *p* value adjustment was made for multiple comparisons.

For each of the nine subgroups evaluated, the reductions in new vertebral and nonvertebral, clinical, and major osteoporotic fracture risk at 43 months were greater in the abaloparatide/alendronate group versus the placebo/alendronate group (Figs. 1 and 2, Supplemental Figs. S1 and S2). Consistent treatment effects were demonstrated, as all point estimates were less than 1.0, except that the hazard ratio of nonvertebral fracture in the lumbar spine BMD *T*‐score > −2.5 subgroup was 1.01. There was one statistically significant (*p* = 0.046) interaction demonstrated for presence versus absence of vertebral fracture at baseline and treatment effect of risk reduction of nonvertebral fracture; the hazard ratios (0.20 and 0.80, respectively) were both less than 1.0. There were two statistically significant interactions with treatment effect demonstrated for major osteoporotic fracture: femoral neck BMD *T*‐score ≤ −2.5 versus > −2.5 (*p* = 0.030) and presence versus absence of a vertebral fracture at baseline (*p* = 0.041). No other statistically significant interactions (all *p* > 0.05) were found among subgroups and fracture prevention treatment effects for any fracture type evaluated.

**Figure 3:**
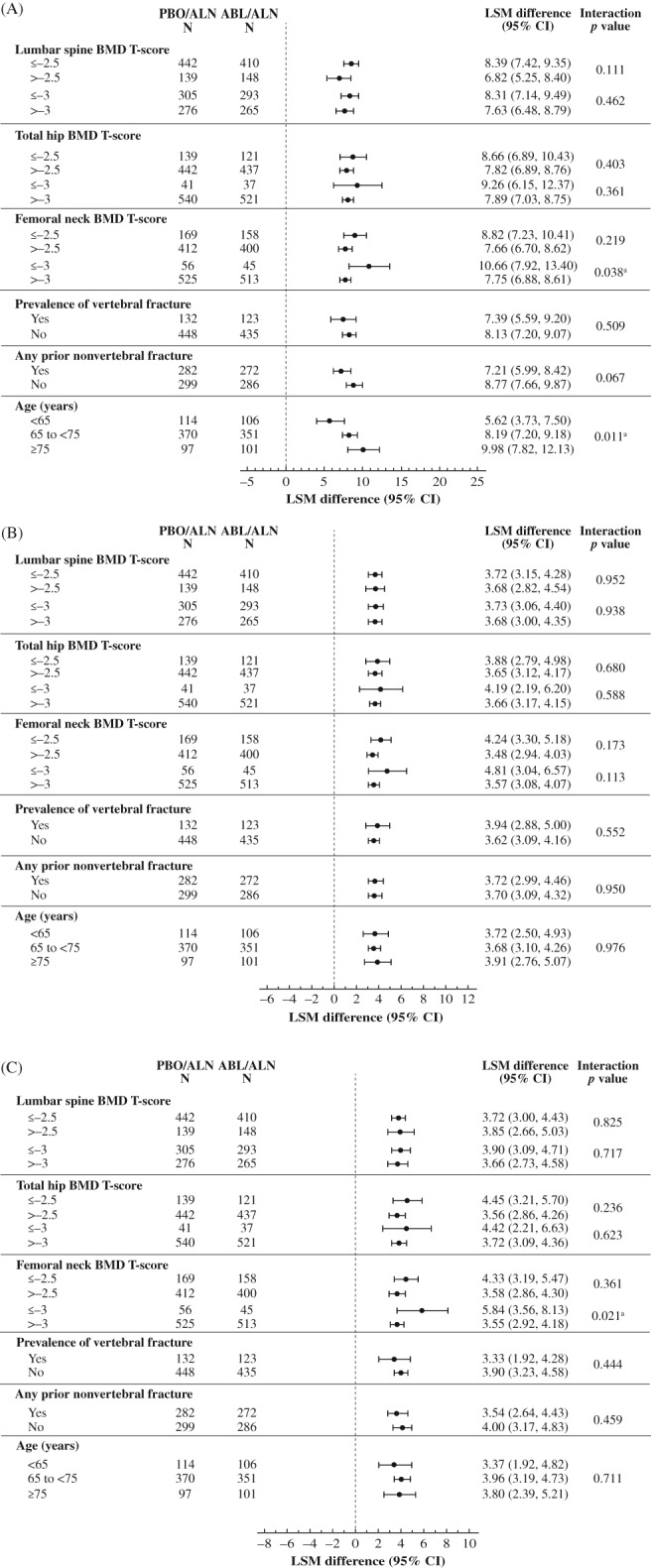
(*A*) Percent change from baseline to month 43 in BMD at the lumbar spine by prespecified subgroup. (*B*) Percent change from baseline to month 43 in total hip BMD by prespecified subgroup. ABL = abaloparatide; ALN = alendronate; BMD = bone mineral density; CI = confidence interval; LSM = least squares mean; PBO = placebo. (*C*) Percent change from baseline to month 43 in BMD at the femoral neck by prespecified subgroup. ABL = abaloparatide; ALN = alendronate; BMD = bone mineral density; CI = confidence interval; LSM = least squares mean; PBO = placebo. ^a^No *p* value adjustment was made for multiple comparisons.

For each of the nine subgroups evaluated, 43‐month lumbar spine BMD improvements were greater in the abaloparatide/alendronate group compared with the placebo/alendronate group (Fig. 3A). Similar results were observed for total hip and femoral neck BMD (Fig. 3 *B*, *C*). There were two statistically significant interactions between subgroups and lumbar spine BMD change: in the femoral neck baseline BMD *T*‐score ≤ −3.0 versus > −3.0 subgroup (*p* = 0.038) and in the age subgroup (*p* = 0.011). There was one significant interaction between subgroups and femoral neck BMD change in the femoral neck baseline BMD *T*‐score ≤ −3.0 versus > −3.0 subgroup (*p* = 0.021). No other statistically significant interactions on change in BMD were found among subgroups and treatment effects.

## Discussion

The significant reductions in fracture risk and BMD improvements associated with abaloparatide in ACTIVE were maintained over the full 43 months of ACTIVExtend in which both groups received up to 24 months of antiresorptive therapy with alendronate after 18 months of abaloparatide or placebo.^5^ In ACTIVExtend, an 84% relative reduction of vertebral fracture risk was observed, and Kaplan–Meier incidence rates of nonvertebral, clinical, and major osteoporotic fractures were significantly lower in the abaloparatide/alendronate group versus the placebo/alendronate group. Likewise, significant gains in BMD achieved during ACTIVE were maintained through the full ACTIVExtend period.

Previous trials have shown that antiresorptive treatment after anabolic therapy preserves the benefits conferred by anabolic therapy^6^, ^7^ and that in the absence of such antiresorptive treatment, the BMD gains are gradually lost.^6^, ^8^ The results of ACTIVExtend demonstrate that the skeletal benefits achieved after 18 months of abaloparatide therapy are extended by subsequent treatment with alendronate, providing further evidence that anabolic therapy followed by an antiresorptive appears to be a compelling strategy to prevent fractures in postmenopausal women with osteoporosis who are at high risk for fracture.^5^

The prespecified subgroup analyses of ACTIVExtend reported here demonstrate that the consistent fracture risk reductions and improvements in BMD observed across baseline risk subgroups at the conclusion of 18 months of abaloparatide treatment were maintained irrespective of baseline risk during 24 months of sequential alendronate treatment. Similar baseline risk subgroup analyses have been performed with other drugs studied as treatments for osteoporosis, including teriparatide,^9^, ^10^ zoledronic acid,^11^ denosumab,^12^ and romosozumab.^13^ In broad terms, each of these analyses demonstrated no clinically important treatment effect differences based on baseline risk subgroup. A previous prespecified analysis of risk factor subgroups in ACTIVE (defined identically as for the analysis reported here) demonstrated similar findings: the efficacy of abaloparatide for fracture risk reduction and BMD gains was consistent across a wide variety of ages and baseline risk, including those with and without prior fractures at study baseline.^3^ Likewise, a prespecified analysis of subgroups defined by geographic region showed that despite geographic variability in baseline fracture risk, there were no differences by region in the effects of abaloparatide for reducing fracture risk across the regions and ethnicities assessed.^14^

The limitations of this analysis are similar to those of other subgroup analyses in which statistical significance was not adjusted for multiple comparisons. ACTIVExtend was powered to detect effects of abaloparatide/alendronate on vertebral fractures in the entire study population rather than in subgroups of the population. In addition, some subgroups were either small or had few fracture events, and these limitations are difficult to compensate for considering the study design.

In conclusion, these analyses confirm that sequential therapy using abaloparatide followed by alendronate appears to be an effective treatment option for a wide range of postmenopausal women at risk for osteoporotic fractures and that the benefits accrued during anabolic treatment with abaloparatide can be maintained, consistently across baseline risk subgroups, for at least 24 months of subsequent antiresorptive treatment.

## Disclosures

BL has received research funding from Amgen and has participated on scientific advisory boards for Amgen and Radius Health, Inc. CZ owns stock in Radius and Amgen and was an employee of Radius Health, Inc., at the time this work was done but is no longer affiliated with Radius Health, Inc. M‐YH owns stock in Radius and was an employee of Radius Health, Inc., at the time of this work but is no longer affiliated with the company. GH owns stock in Radius Health, Inc., was an employee of Radius Health, Inc., at the time this work was done, and is a member of the Scientific Advisory Board at Radius Health, Inc. RKD is a speaker for Radius Health, Inc., Amgen, and Eli Lilly and is a consultant to Amgen and Eli Lilly. NEL is a consultant for Amgen and Radius Health, Inc., and has participated in advisory boards for Amgen and Radius Health, Inc. AJS has received research funding from Radius Health, Inc., participated on scientific advisory boards or been a consultant for Agnovos, Amgen, Eli Lilly, Merit Medical, Radius Health, Inc., and UCB, and is a speaker for Amgen, Eli Lilly, and Radius Health, Inc.

## Supporting information

Supplemental **Fig. S1**. Clinical fracture risk reduction for abaloparatide/alendronate versus placebo/alendronate by prespecified subgroup. ABL = abaloparatide; ALN = alendronate; BMD = bone mineral density; CI = confidence interval; HR = hazard ratio; PBO = placebo.

Supplemental **Fig. S2**. Major osteoporotic fracture risk reduction for abaloparatide/alendronate versus placebo/alendronate by prespecified subgroup. ABL = abaloparatide; ALN = alendronate; BMD = bone mineral density; CI = confidence interval; HR = hazard ratio; PBO = placebo. ^a^No *p* value adjustment was made for multiple comparisons.
